# The influence of oxygen and oxidative stress on *de novo* acquisition of antibiotic resistance in *E. coli* and *Lactobacillus lactis*

**DOI:** 10.1186/s12866-023-03031-4

**Published:** 2023-10-02

**Authors:** Wenxi Qi, Martijs J. Jonker, Lisa Teichmann, Meike Wortel, Benno H. ter Kuile

**Affiliations:** 1 Laboratory for Molecular Biology and Microbial Food Safety, Swammerdam Institute for Life Sciences, University of Amsterdam, Amsterdam, The Netherlands; 2 RNA Biology & Applied Bioinformatics, Swammerdam Institute for Life Sciences, University of Amsterdam, Amsterdam, The Netherlands; 3 Netherlands Food and Consumer Product Safety Authority, Office for Risk Assessment, Utrecht, The Netherlands

**Keywords:** Reactive oxygen species, *De novo* resistance, Antimicrobial resistance, Whole genome sequencing, Reactive metabolic byproducts

## Abstract

**Background:**

Bacteria can acquire resistance through DNA mutations in response to exposure to sub-lethal concentrations of antibiotics. According to the radical-based theory, reactive oxygen species (ROS), a byproduct of the respiratory pathway, and oxidative stress caused by reactive metabolic byproducts, play a role in cell death as secondary killing mechanism. In this study we address the question whether ROS also affects development of resistance, in the conditions that the cells is not killed by the antibiotic.

**Results:**

To investigate whether oxygen and ROS affect *de novo* acquisition of antibiotic resistance, evolution of resistance due to exposure to non-lethal levels of antimicrobials was compared in *E. coli* wildtype and Δ*oxyR* strains under aerobic and anaerobic conditions. Since *Lactococcus lactis* (*L. lactis*) does not have an active electron transport chain (ETC) even in the presence of oxygen, and thus forms much less ROS, resistance development in *L. lactis* was used to distinguish between oxygen and ROS. The resistance acquisition in *E. coli* wildtype under aerobic and anaerobic conditions did not differ much. However, the aerobically grown Δ*oxyR* strain gained resistance faster than the wildtype or anaerobic Δ*oxyR.* Inducing an ETC by adding heme increased the rate at which *L. lactis* acquired resistance. Whole genome sequencing identified specific mutations involved in the acquisition of resistance. These mutations were specific for each antibiotic. The *lexA* mutation in Δ*oxyR* strain under aerobic conditions indicated that the SOS response was involved in resistance acquisition.

**Conclusions:**

The concept of hormesis can explain the beneficial effects of low levels of ROS and reactive metabolic byproducts, while high levels are lethal. DNA repair and mutagenesis may therefore expedite development of resistance. Taken together, the results suggest that oxygen as such barely affects resistance development. Nevertheless, non-lethal levels of ROS stimulate *de novo* acquisition of antibiotic resistance.

## Background

Bacteria can acquire antibiotic resistance by adapting cellular physiology, DNA mutations, and horizontal transfer of resistance genes [1]. DNA mutations can occur as a result of exposure to non-lethal concentrations of antimicrobials [2]. These mutations can modify the cellular targets of antibiotics, activate antibiotic efflux pumps, generate enzymes that disable antibiotics, and reduce the permeability of membranes to antibiotics to make bacteria resistant [3, 4]. While the role of mutations in development of *de novo* resistance has been documented to some extent [5–7], the driving factors for mutations in the bacterial DNA are less well described.

According to the radical-based theory, exposure to bactericidal antibiotics results in the formation of reactive oxygen species (ROS) and reactive metabolic byproducts as a secondary effect that hastens bacterial cell death [8–10]. The overproduction of ROS damages the DNA, proteins, lipids, and nucleotides pool, and in particular causes the oxidation of guanine to 8-oxo-guanine [11]. Besides being lethal, ROS can also enhance mutation rates [12]. In fact, the overall effect of ROS and reactive metabolic byproducts may be hormetic [13], as low concentrations enable rapid adaptation, while high level are lethal [14]. Sub-inhibitory doses of ciprofloxacin generate a resistant mutant subpopulation through ROS formation and sigma-S general stress response activity [15]. Furthermore, multidrug resistance induced by sublethal levels of antibiotics correlates with ROS-induced mutagenesis [16]. ROS has been described as a key factor in antibiotic-induced SOS mutagenesis, and treatment with the antioxidant N-acetylcysteine reduces ROS and blocks SOS-mediated mutagenesis [17]. Based on the above considerations we hypothesized that the oxidative stress caused by ROS plays a central role in *de novo* acquisition of antibiotic resistance.

*Escherichia coli* is a facultative anaerobic prokaryote, commonly found in the human gastrointestinal tract [18]. The gene *oxyR* in *E. coli* codes for an oxidative stress regulator, mitigating levels of hydrogen peroxide under aerobic conditions [19], and *oxyR* is involved in preventing SOS-induced DNA damage by hydrogen peroxide [20] Additionally, *oxyR* regulon mutants of *E. coli* and *Salmonella typhimurium* were associated with antibiotic resistance [21]. Because *E. coli* is well described and thoroughly studied in many aspects, it is the primary model organism used in this study. The lactic acid bacterium *Lactococcus lactis* is a fermentative bacterium, that can also grow in the presence of oxygen, but even then does not possess a complete electron transport chain (ETC) [22]. However, when both heme and oxygen are present, *L. lactis* can establish an ETC, resulting in NADH oxidation and aerobic respiration [23] and hence the formation of ROS. It is therefore used throughout this study to separate the respective roles of oxygen and ROS generated by the ETC in development of *de novo* antimicrobial resistance.

The information available in the scientific literature as summarized above lead us to formulate the following hypothesis: ROS possibly is a driving factor for *de novo* acquisition of resistance in bacteria surviving exposure to bactericidal antibiotics. The effect of ROS is likely to be hormetic, in the sense that low levels of stress caused by bactericidal antimicrobials is beneficial for the cell as resistance is acquired. High levels, however, are lethal. This hypothesis was examined by documenting development of resistance under aerobic and anaerobic conditions, in wildtype *E. coli* and the Δ*oxyR* mutant *E. coli* that has reduced ability to remove ROS [24]. *L. lactis* was used as a biological control to separate between the effects of oxygen itself and ROS.

## Results

### Acquisition of resistance under aerobic and anaerobic conditions

**Fig. 1:**
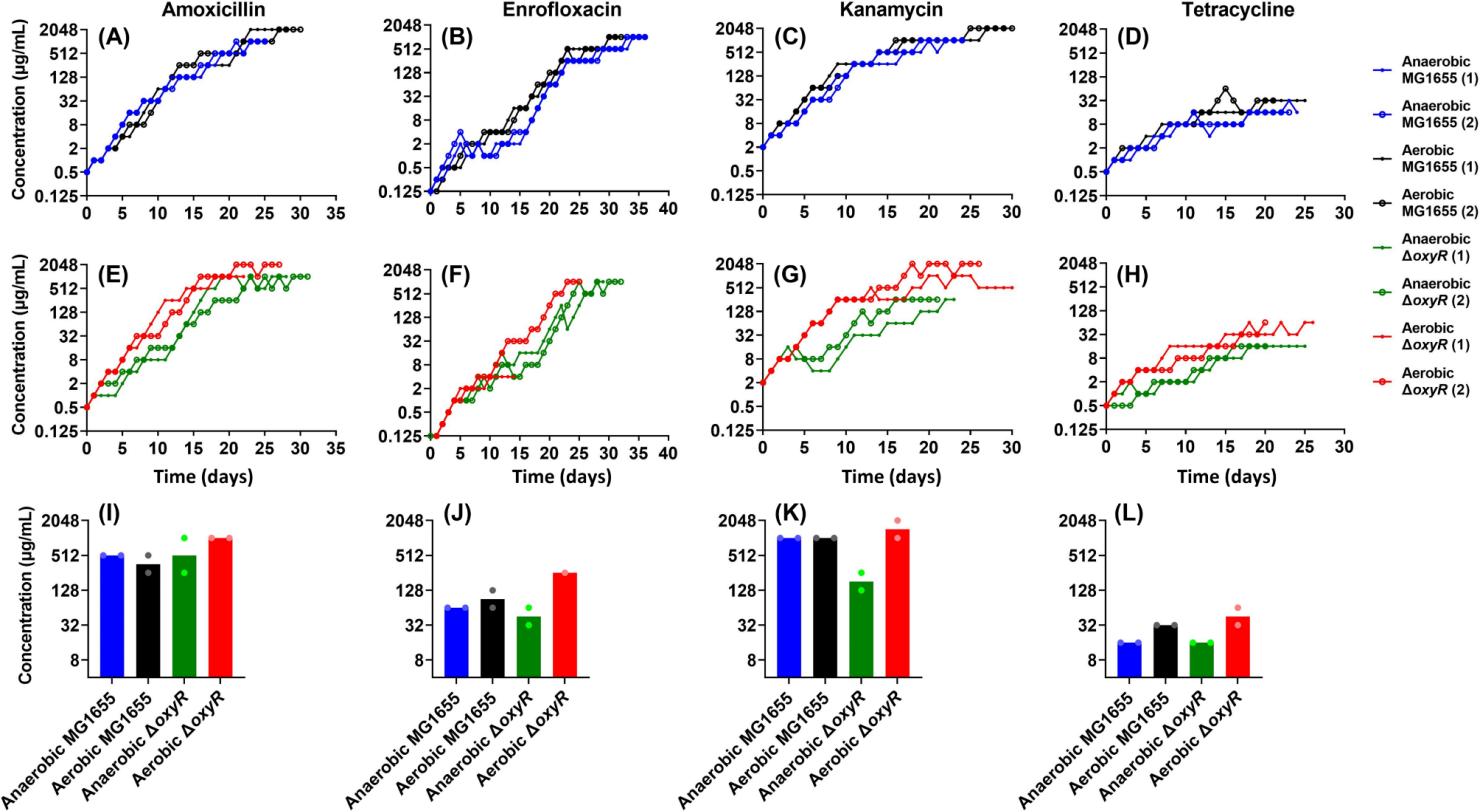
Acquisition of resistance to amoxicillin (**A**, **E**, **I**), enrofloxacin (**B**, **F**, **J**), kanamycin (**C**, **G**, **K**), and tetracycline (**D**, **H**, **L**) of *E. coli* wild-type MG1655 (blue: anaerobic/black: aerobic) and the Δ*oxyR* knockout (green: anaerobic/red: aerobic) strains. The Y-axis indicates the antibiotic concentration at which the cells were able to grow. The top 4 panels (**A**-**D**) show wild-type MG1655, the second set of panels (**E**-**H**) show the Δ*oxyR* mutant, and the third set of panels (**I**-**L**) compares the resistance concentrations of each strain reached at day 20

In order to evaluate the effect of oxygen on the development of resistance by *E. coli* exposed to sub-lethal levels of antibiotics, initially fully susceptible cells were grown in the presence of stepwise increasing concentrations of four antimicrobials. The evolution of resistance defined as the ability to grow at certain concentrations of the antibiotics to the bactericidal antibiotics amoxicillin, enrofloxacin, kanamycin, and the bacteriostatic antibiotic tetracycline under aerobic and anaerobic conditions was compared (Fig. 1). To provide additional insight into the role of cellular systems that induce stress caused by reactive oxygen species (ROS), two *E. coli* strains were used in these experiments: The wild-type MG1655 and the Δ*oxyR* single gene deletion strain derived from it. OxyR is a transcriptional dual regulator of antioxidant gene expression in response to oxidative stress. Hence, the cell will produce more ROS when *oxyR* is knocked out [24]. In approximately 30 days, resistance against bactericidal antibiotics (amoxicillin, enrofloxacin, and kanamycin) reached a high concentration (512–2048 µg/mL), while resistance against the bacteriostatic tetracycline was limited to around 32 µg/mL (Fig. 1).

There were only minor differences in the final concentrations reached by the wild-type MG1655 under aerobic or anaerobic incubations. The only clear difference was that the final concentration for amoxicillin and kanamycin was double after aerobic growth compared to anaerobic (Fig. 1A and C). In the case of the Δ*oxyR* mutant, the aerobic incubations reached higher resistance levels and reached them faster, especially in the case of bactericidal antibiotics (Fig. 1E-G). After 20 days, the resistance concentrations reached showed major differences. The Δ*oxyR* mutant strain under aerobic conditions reached to the highest concentrations compared to the other strains (Fig. 1I-L).

### Acquisition of resistance in *E. coli* and *L. lactis* in rich medium

To separate the influence of oxygen itself from that of oxygen derived compounds, such as ROS, the experiments were repeated on *L. lactis*. As one of very rare microbes *L. lactis* is homofermentative, both under aerobic and anaerobic conditions, because it lacks the pathway for heme biosynthesis and hence cannot form an endogenous ETC. However, an active respiratory chain is established when the culture is grown in the presence of heme. These features make *L. lactis* an ideal model organism to address the question of the influence of different levels of activity of the ETC on the development of antibiotic resistance. As *L. lactis* can only be cultured in rich medium, the experiments were repeated for *E. coli* grown in rich LB medium as well to facilitate the comparison.

**Fig. 2:**
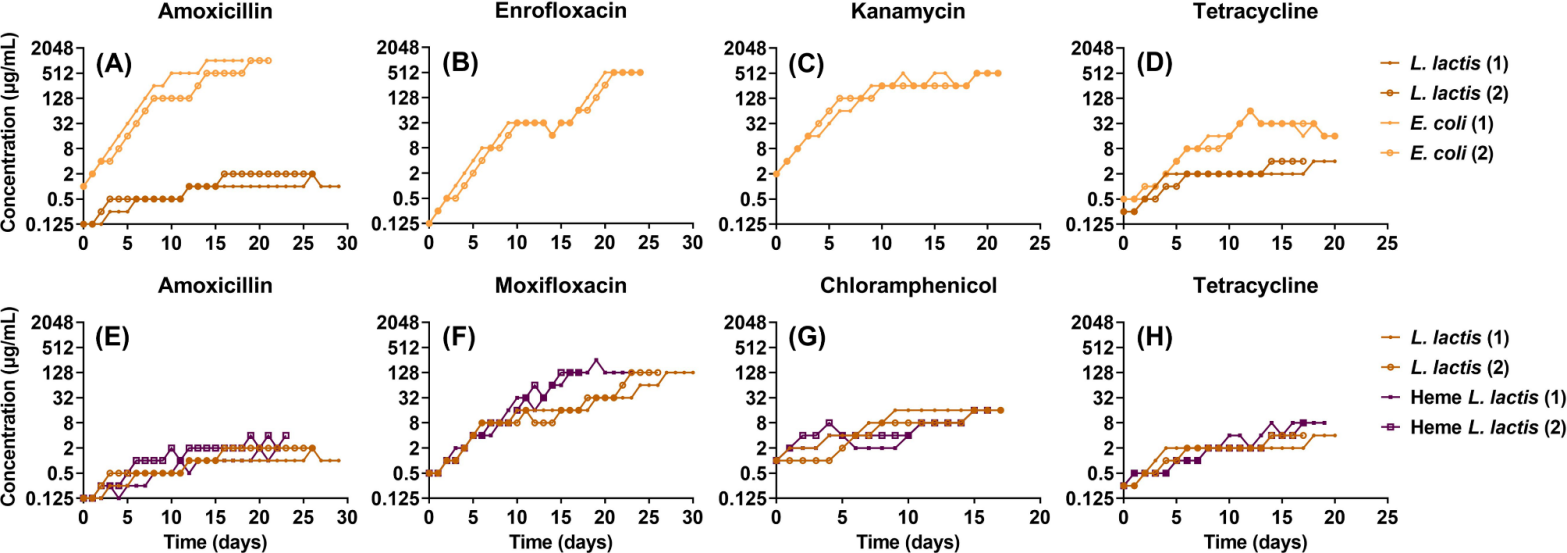
Acquisition of resistance by *E. coli* MG1655 to amoxicillin (**A**), enrofloxacin (**B**), kanamycin (**C**), tetracycline(**D**) in rich medium (LB), and *L. lactis* MG1363 to amoxicillin (**A**), tetracycline (**D**) in rich medium (M17). Acquisition of resistance to amoxicillin (**E**), moxifloxacin (**F**), chloramphenicol (**G**), and tetracycline (**H**) by *L. lactis* and Heme-added *L. lactis* in M17 medium

After 30 days the resistance to amoxicillin was still in the low range of concentrations (1 or 2 µg/mL) in *L. lactis*, compared to 1024 µg/mL in *E. coli* (Fig. 2A). Because *L. lactis* is intrinsically resistant to enrofloxacin and kanamycin, these were omitted (Fig. 2B and C). Instead, moxifloxacin and chloramphenicol were used. During the tetracycline resistance development, tolerated antibiotic concentrations increased from 0.25 µg/mL to 4 µg/mL (16-fold) in *L. lactis*, from 0.5 µg/mL to 16 µg/mL (32-fold) in *E. coli* (Fig. 2D). *L. lactis* barely acquired amoxicillin resistance even with added heme (Fig. 2E). Only in the case of moxifloxacin resistance did the addition of heme make a noticeable difference (Fig. 2F), but not on the evolution of resistance to the bacteriostatic antibiotics: chloramphenicol and tetracycline (Fig. 2G and H).

### ROS production levels in antibiotic resistant strains

**Fig. 3:**
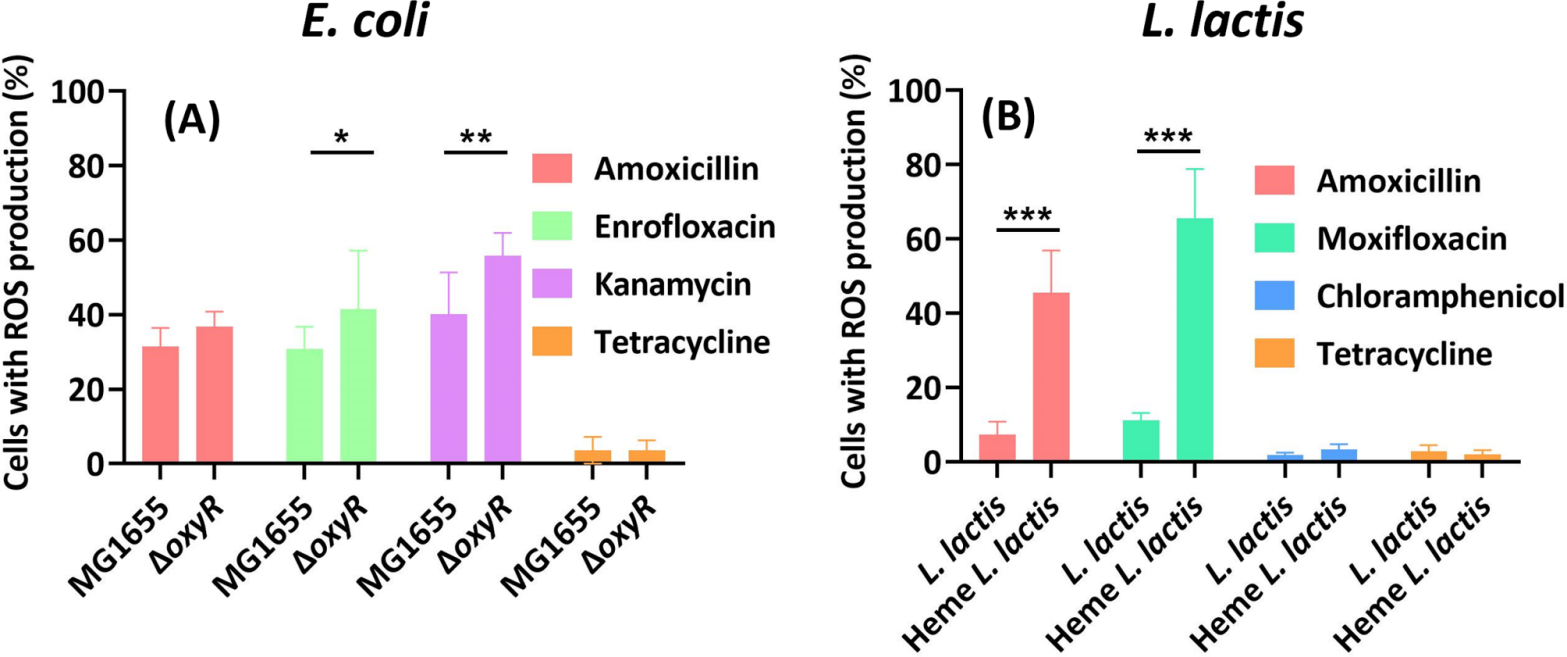
ROS measurement in antibiotic resistant *E. coli* and *L. lactis* under fluorescent microscopy. Resistant strains were treated with the highest concentrations of antibiotics that still allowed growth. The fluorescent dye H_2_DCFDA was used to detect the ROS. Cells with ROS production were counted with ImageJ, *E. coli* (**A**), *L. lactis* (**B**). Means ± SD, statistical significance was investigated using a one-way ANOVA *p < 0.05, **p < 0.001, ***p < 0.0001

The formation of ROS was measured by fluorescent microscopy. Strains of *E. coli* were made resistant to the amoxicillin, enrofloxacin, kanamycin and tetracycline. On exposure to these antibiotics, cells exposed to the bactericidal antibiotics had higher ROS production than strains grown in the presence of the bacteriostatic tetracycline (Fig. 3A). Compared to the MG1655 wild type, the generation of ROS in Δ*oxyR* strains was significantly enhanced in the presence of enrofloxacin and kanamycin. In *L. lactis*, only heme added strains that were exposed to the bactericidals amoxicillin and moxifloxacin had noticeable ROS production (Fig. 3B). In the presence of the bacteriostatic antibiotic chloramphenicol and tetracycline, almost no ROS was generated in *L. lactis* whether heme was added or not. These observations indicate that exposure to bactericidal antibiotics increases ROS production in the *oxyR* knockout to higher levels than in wildtype *E. coli*, and similarly in heme added *L. lactis* compared to *L. lactis* growing in regular medium without heme.

### Whole genome sequencing to document mutations during resistance evolution

**Fig. 4:**
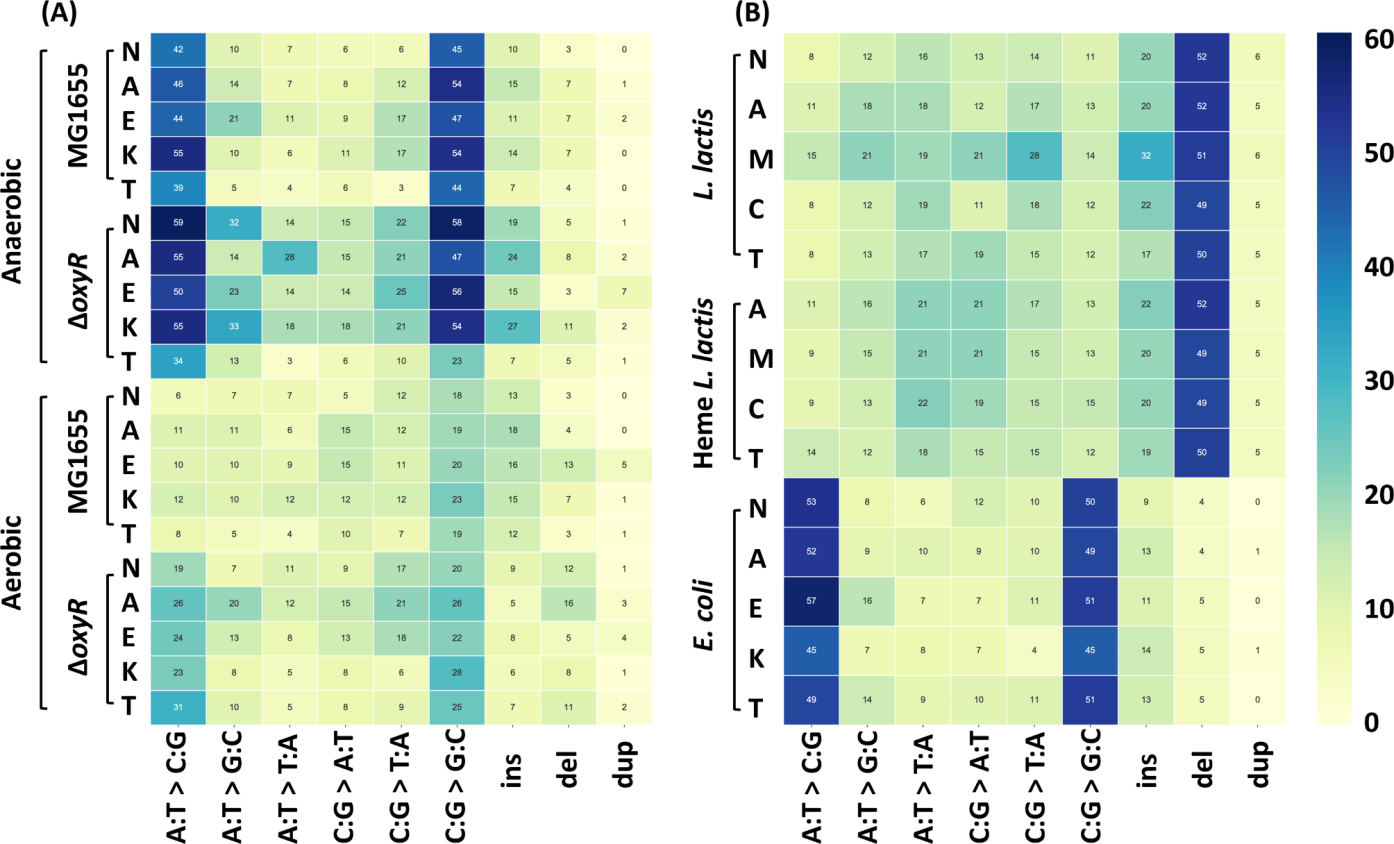
Heat map of the types of nucleotide changes in MG1655 and Δ*oxyR* under aerobic and anaerobic conditions. The color codes for the number of specified mutations observed. (**A**); *L. lactis*, heme-added *L. lactis* and *E. coli* in rich medium (**B**). N: no treatment control, A: amoxicillin, E: enrofloxacin, K: kanamycin, T: tetracycline, M: moxifloxacin, C: chloramphenicol. Ins: insertion, del: deletion, dup: duplication

**Fig. 5:**
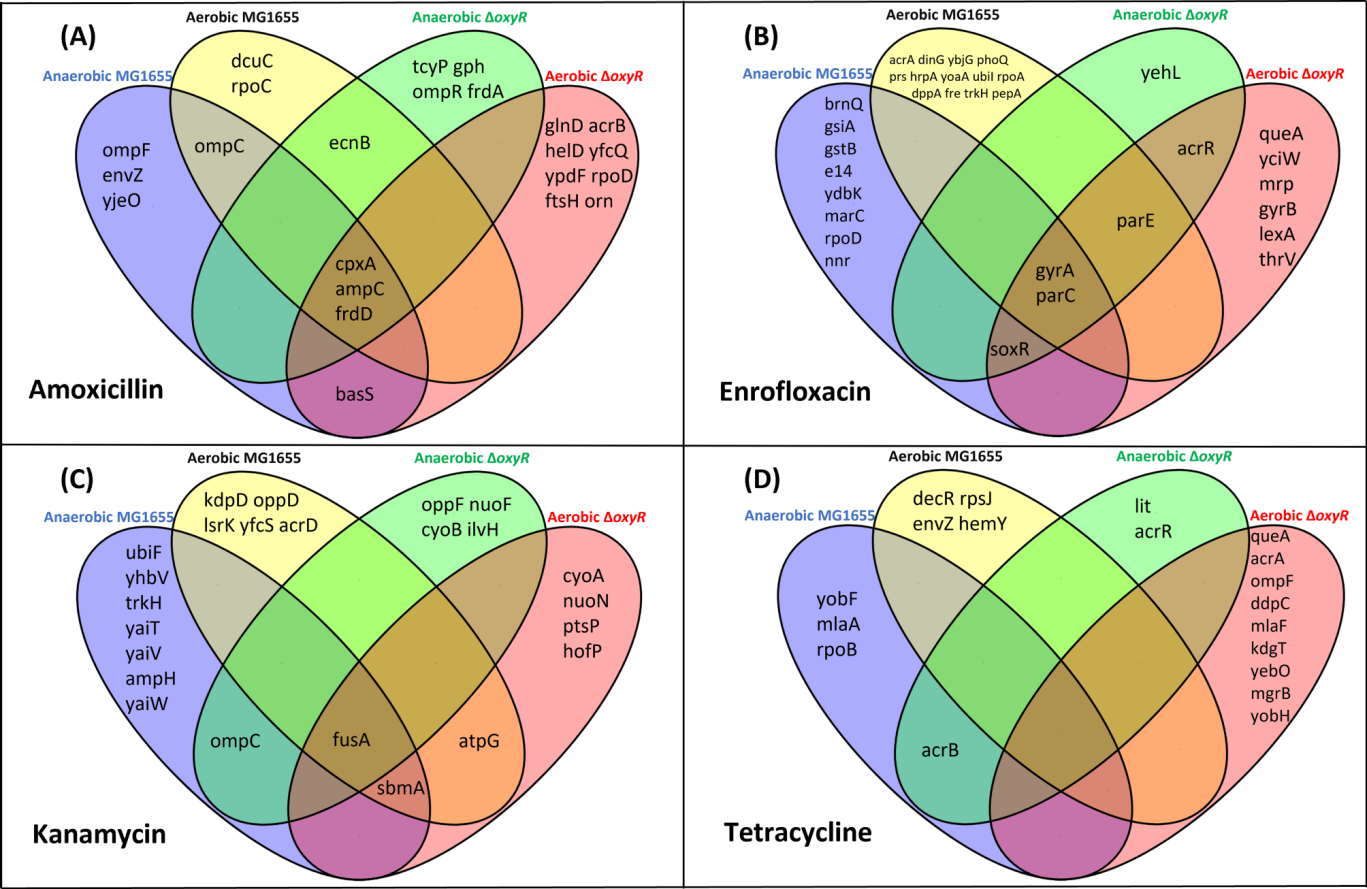
Overlap of mutated genes in the anaerobic wild-type MG1655 (blue oval), the aerobically grown wild-type MG1655 (yellow oval), the anaerobic mutant Δ*oxyR* (green oval), and the aerobic mutant Δ*oxyR* (red oval) made resistant to amoxicillin (**A**), enrofloxacin (**B**), kanamycin (**C**), and tetracycline(**D**)

**Fig. 6:**
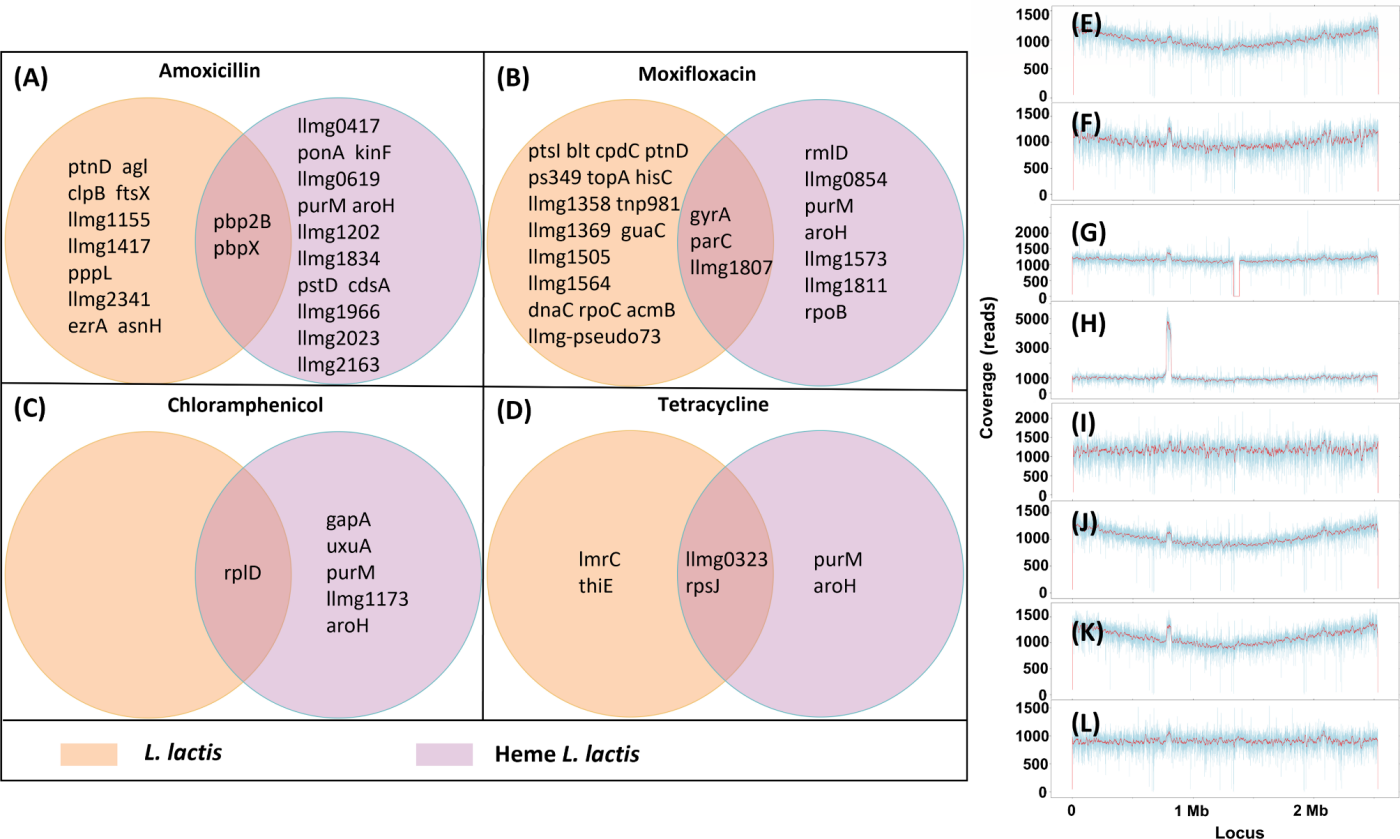
Mutated genes in wild-type *L. lactis* and heme-added *L. lactis* after evolution of resistance to amoxicillin (**A**), moxifloxacin (**B**), chloramphenicol (**C**), and tetracycline (**D**). DNA copy number of WGS of *L. lactis* resistant to amoxicillin (**E**, **F**), moxifloxacin (**G**, **H**), chloramphenicol (**I**, **J**), and tetracycline (**K**, **L**) in no heme-added *L. lactis* (**E**, **G**, **I**, **K**) is compared to heme-added *L. lactis* (**F**, **H**, **J**, **L**)

**Fig. 7:**
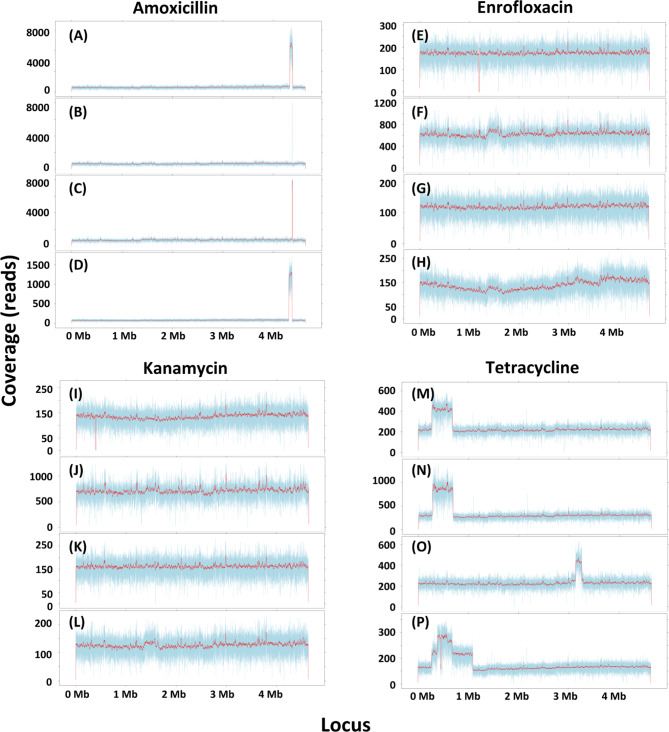
Variation of DNA copy numbers in whole genome sequencing (WGS) over the entire genome of the anaerobically grown wild-type MG1655 (**A**, **E**, **I**, **M**), aerobic wild-type MG1655 (**B**, **F**, **J**, **N**), anaerobic mutant Δ*oxyR* (**C**, **G**, **K**, **O**), and aerobic mutant Δ*oxyR* (**D**, **H**, **L**, **P**) made resistant to amoxicillin (**A**-**D**), enrofloxacin (**E**-**H**), kanamycin (**I**-**L**), and tetracycline(**M**-**P**)

To identify mutations that accompany the acquisition of resistance, the genomic DNA of the strains resistant to the highest antibiotic concentrations was sequenced entirely. The strains to be sequenced were the final incubations of the evolution experiments. In case the two replicates did not have an identical MIC, the most resistant strain was selected. Variant calling analysis was used to document nucleotide changes as summarized in Fig. 4. The most frequently observed types of nucleotides changes were AT to CG and CG to GC (Fig. 4). Higher numbers of these two types were detected under anaerobic conditions than under aerobic conditions. The MG1655 strain in aerobic conditions had the fewest mutations. *L. lactis* mainly acquired deletion mutations less of 20 bp (Fig. 4). After excluding the same mutations observed in strains grown without antibiotics, the unique mutated genes in each strain were identified and summarized in Venn diagram (Figs. 5 and 6A-D). DNA copy number variations are shown in Fig. 7; Table 1, and Fig. 6E-L.

**Table 1:** Amoxicillin resistant strains’ amplification contigs. Fragments of the chromosomal DNA that all include the *ampC* gene but vary in length, are multiplied by the indicated factor

Strains	Length (kb)	Copy numbers (times)
Anaerobic MG1655	53	30
Aerobic MG1655	2.5	17
Anaerobic Δ*oxyR*	5.5	41
Aerobic Δ*oxyR*	59	9

In all amoxicillin resistant strains, the genes *ampC*, *frdD*, and *cpxA* were mutated (Fig. 5A). AmpC is the serine beta-lactamase with substrate specificity for amoxicillin, FrdD is the fumarate reductase subunit D [25]. CpxA is a membrane-localized sensor kinase that activates CpxR, which promotes efflux complex expression [26]. The four resistant strains all contained a differential amplification contig that included *ampC* and *frdD* (Fig. 7A-D; Table 1). In the resistant strains, some outer membrane porin genes such as *ompF* and *ompC* were mutated. These are associated with reduced permeability to antibiotics. Only the Δ*oxyR* strain under aerobic conditions had a mutation in *acrB*, which codes for an efflux pump.

Strains made resistant to enrofloxacin shared two common mutated genes, DNA gyrase gene, *gyrA*, and DNA topoisomerase gene, *parC*, which are well-known quinolone resistance genes [27] (Fig. 5B). Other typical quinolone resistance related genes, *gyrB* and *parE* were mutated in the Δ*oxyR* strain under aerobic conditions, *parE* was mutated as well in the Δ*oxyR* strain under anaerobic conditions and the MG1655 strain under aerobic conditions. Several DNA or RNA helicase genes were mutated, such as *dinG*, *hrpA*, and *yoaA*. Efflux pump associated genes like *acrA*, *phoQ* were mutated in the MG1655 strain under aerobic conditions, and *acrR*, *soxR* in the Δ*oxyR* strains both under aerobic and anaerobic conditions. The MG1655 strain under anaerobic conditions contained a cryptic prophage e14 deletion (Fig. 7E), which is a well-known mutation associated with quinolone resistance [28]. In *lexA*, which inhibits a number of genes involved in the SOS response to DNA damage [29, 30], a Gly85Ser mutation was observed in the Δ*oxyR* strain under aerobic conditions.

The common mutated gene in kanamycin resistant strains is *fusA* (Fig. 5C). During the translation elongation, FusA catalyzes the GTP-dependent ribosomal translocation step [31]. This mutation may cause antibiotic target alteration. The MG1655 strain under anaerobic conditions had a 5 kb deletion from gene *yaiT* to *yaiW*. (Fig. 7I). This deletion includes *sbmA*, a peptide antibiotic transporter. The *sbmA* mutation also occurred in both the MG1655 and Δ*oxyR* strains under aerobic conditions.

There was no common mutated gene in the tetracycline resistant strains (Fig. 5D). However, a few resistance-related mutated genes were identified. For instance, genes that code for the antibiotic efflux pumps, such as *acrA*, *acrB*, *acrR*, *mlaF* were mutated. Genes that associated with antibiotic target alteration or protection, like *rpoB* and *rpsJ*; and genes associated with reduced permeability to antibiotics, such as *ompF* were also mutated. The MG1655 strains under aerobic and anaerobic conditions both contained a 2-fold amplification region from *insH1* to *insH3* of around 400 kb length (Fig. 7M and N). The amplified region in the Δ*oxyR* strain under aerobic conditions started from *insH1* as well but stopped in *insF1* resulting in a total length of about 800 kb (Fig. 7P). A roughly 100 kb amplification region was observed in the Δ*oxyR* strain under anaerobic conditions from gene *insH1* to *yahH* (Fig. 7O).

### Mutations in *L. lactis* with and without added heme

In *L. lactis*, the number of mutated genes in strains made resistant to bactericidal antibiotics was higher than in strains resistant to bacteriostatic antibiotics (Fig. 6). When heme was added to generate a functional ETC *L. lactis* strains that evolved resistance contained mutations in two genes, *purM* (Gly250Val), and *aroH* (Arg168Gly). PurM is involved in purine metabolism [32]. AroH is involved in the early step of aromatic amino acid biosynthesis [33]. In amoxicillin resistant *L. lactis*, the mutated genes, *pbp2B* and *pbpX* were observed both with and without added heme. Mutations in these two genes may cause antibiotic action targets modification. The target genes *ponA* and *cdsA* were mutated in the heme-added strain as well (Fig. 6A).

The common mutated genes in moxifloxacin resistant strains were *gyrA*, *parC*, and *llmg1807*. The genes *rpoC* were mutated in the no heme-added condition, and *rpoB* in heme-added condition (Fig. 6B). There was a roughly 45 kb length deletion from *llmg1359* to *llmg1411* in no heme-added strain (Fig. 6G). A 42 kb amplification, with around 4-fold copy number increase, from *ps301* to *ps357* occurred in the heme-added moxifloxacin resistant strain (Fig. 6H). Only one mutated gene, *rplD* (Lys68Asn) was detected in no heme-added chloramphenicol resistant *L. lactis*. RplD, the 50 S L4 ribosomal protein, was identified in *Neisseria gonorrhoeae* as a macrolide resistance protein [34]. The same gene *rplD* was mutated but with different amino acid change, Lys68Glu, in the heme-added chloramphenicol resistant *L. lactis*. The tetracycline resistant *L. lactis* strains had two common mutated genes, *llmg0323* and *rpsJ*. Llmg0323 is a transcriptional regulator. RpsJ, 30 S ribosomal protein S10 is a tetracycline-resistant ribosomal protection protein [35].

### Functional annotation of mutated genes associated with antibiotic resistance

**Fig. 8:**
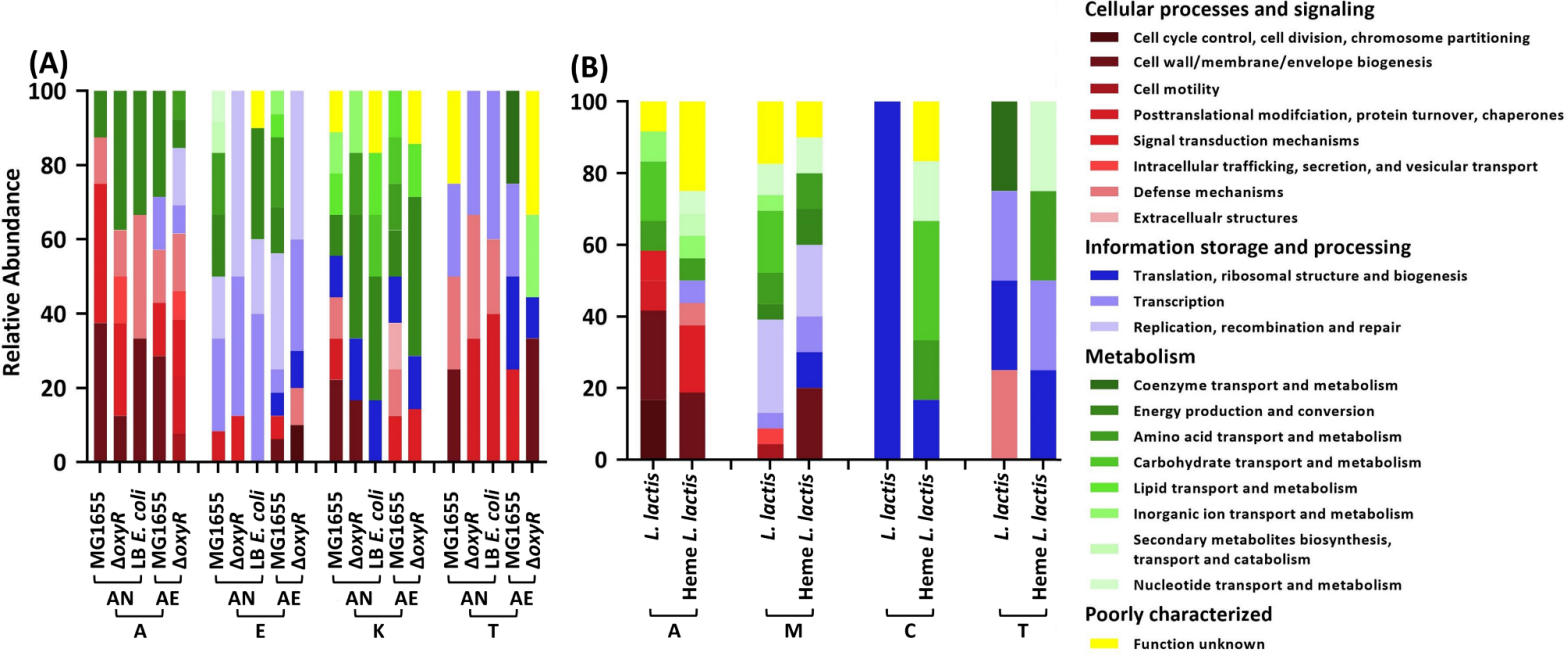
Cluster of orthologous groups (COG) classifications of mutated genes. A: amoxicillin, E: enrofloxacin, K: kanamycin, T: tetracycline, M: moxifloxacin, C: chloramphenicol; AN: anaerobic condition, AE: aerobic condition. Panel **A**: *E. coli*; Panel **B**: *L. lactis*. The IMG/M was used to classify the genes (https://img.jgi.doe.gov/)

In order to group the mutated genes, they were clustered functionally according to the phylogenetic classification (Fig. 8). The IMG/M database of Clusters of Orthologous Groups (COG) was used to annotate all the mutated genes and classify them [36]. There were no clear differences in which classes of genes were mutated between *E. coli* wild-type MG1655 and the Δ*oxyR* strains under aerobic and anaerobic conditions (Fig. 8A). However, the different antibiotics caused mutations in different classes of genes. The majority of genes mutated after exposure to amoxicillin, enrofloxacin, and kanamycin are involved in cellular processing and signaling, information storage and processing, and metabolism, respectively (Fig. 8A). In *L. lactis*, half the number of mutated genes in amoxicillin exposed strains is involved in cellular processes and signaling function. The genes mutated by growth in the presence of moxifloxacin were evenly distributed over information and signaling and metabolism (Fig. 8B). The number of mutated genes in tetracycline-incubated *E. coli*, and chloramphenicol, tetracycline-incubated *L. lactis* were clearly lower than those of cells exposed to bactericidal antibiotics, making the COG comparison problematic.

## Discussion

The role of oxygen in the killing of bacteria by bactericidal antibiotics has been the subject of debate since the “radical based” theory was proposed, which suggests that reactive oxygen species form a secondary killing mechanism in addition to the primary target [8, 37, 38]. This study examined the role of oxygen in *de novo* acquisition of resistance by comparing resistance development under aerobic and anaerobic conditions. In the human intestines, *E. coli* is exposed to anaerobic or micro-aerobic conditions [39]. Anaerobic growth may therefore be more natural than the aerobic conditions used in most experiments involving *E. coli*. To test the influence of oxidative stress, resistance adaptation was examined in an *E. coli* Δ*oxyR* mutant strain. *L. lactis* was used to separate between the effects of oxygen and those of ROS, because even under aerobic conditions, it does not possess a complete ETC, unless heme is supplied in the medium [40]. Hence, it only forms ROS when heme is present in the medium, but not in the absence, even when oxygen is available.

### Hormesis

The *de novo* acquisition of resistance in wildtype *E. coli* with and without oxygen was relatively similar. The presence of oxygen in itself seems to have no major influence on resistance development. However, under anaerobic conditions, reactive metabolic byproducts (RMB), particularly reactive electrophilic species, accumulate in antibiotic-treated *E. coli* [10]. These RMB contribute to cell death in a similar manner as ROS in aerobic conditions. Possibly ROS and RMB have relatively similar effects on resistance development. Nonetheless, under aerobic conditions, Δ*oxyR* strain became resistant to bactericidal antimicrobials faster than the wildtype. The *oxyR* gene codes for a regulatory protein that regulates a system of proteins that protect the cell from ROS and comparable stressors [41]. The presence of oxygen accelerated the acquisition of resistance in the Δ*oxyR* strains, indicating that the extra ROS indeed stimulated this process. Hyperproduced ROS damage DNA and the nucleotide pool. The 8-oxo-guanine derived from guanine results in faulty DNA replication, accompanied by the general stress response [42, 43]. The cell’s repair systems tend to increase rates of mutagenesis [44].

During antibiotic resistance acquisition, the differences can be observed in the middle and late stages of resistance development, possibly because at that stage increasing numbers of cells accumulate general stress-induced mutations. The observation that heme-added *L. lactis* gained moxifloxacin resistance faster also points to an effect of ROS (Fig. 2F), generated by the ETC that was induced by the addition of heme. These observations can be understood in the framework of hormesis [45, 14]. While mild stress is beneficial in the form of the ability to more rapidly adapt to the presence of antimicrobials, high levels of the same stressors can cause cell death. When radical-based stress levels are low, a relatively moderate increase of the oxidative stress seems to accelerate the acquisition of resistance. This modest amount of oxidative stress often occurs in environmental microbes and correlates well with antibiotic-resistance gene abundance [46].

### Mutations accompanying resistance development

Far higher mutation frequencies of AT to CG and CG to GC were observed in anaerobically grown *E. coli* than in aerobic conditions. A likely cause is the DNA damage induced by acidic fermentation [47, 48]. However, these kinds of single nucleotide change mutations and the < 20 bp deletion mutations which occurred in the *L. lactis* were almost never situated in resistance-related genes hot spots. Anaerobically grown untreated *E. coli* strains also had high mutation frequencies, indicating that this kind of mutation did not influence resistance development.

Various *E. coli* strains which evolved resistance to the bactericidal antibiotics had common mutated genes, for instance, *ampC* for amoxicillin, *gyrA* and *parC* for enrofloxacin, and *fusA* for kanamycin. These genes are known to play crucial roles in resistance acquisition [2]. In addition to the mutated genes that are common to all strains with induced resistance, there are mutations in genes that are specific for a certain strain and that follow logically from the characteristics of that strain. Only the amoxicillin resistant *E. coli* Δ*oxyR* strain under aerobic conditions had an *acrB* mutation that may increase amoxicillin efflux. In the aerobically grown Δ*oxyR* enrofloxacin resistant strain, a mutation was observed in *lexA*. *lexA* codes for a transcriptional repressor, auto-cleavage of LexA triggers the SOS response [49]. Mutagenic states caused by the SOS stress response may enhance the *de novo* acquisition of antibiotic resistance [50, 51]. The Gly85Ser mutation located on the *lexA* auto-cleavage site [52], may increase the rate of acquisition of resistance. During kanamycin exposure, SbmA mutations included Phe6fs in the aerobically grown wildtype strain, total deletion in the anaerobically grown wildtype, and Ser372* in the aerobically grown Δ*oxyR* strain. These mutations block SbmA activity, thus inhibiting kanamycin uptake [53]. Three of the four cell lines exposed to kanamycin acquired mutations in *sbmA* that reduced kanamycin uptake. Only the anaerobically grown Δ*oxyR* strain missed a *sbmA* mutation, and indeed developed less kanamycin resistance.

Mutations in the multidrug efflux pump subunit coding genes *acrA*, *acrB*, *acrD*, and their regulator *acrR*, emerged in four antibiotic-resistant *E. coli* strains. Additionally, mutations in the outer membrane porin genes *envZ* (*ompB*), *ompC*, and *ompF* appeared in the amoxicillin, kanamycin, and tetracycline resistant *E. coli* strains. This could lead to increased resistance when these strains are exposed to other antibiotic treatments due to the cross-resistance [54]. Comparison with a similar dataset in which *E. coli* wildtype evolved resistance resistant against the same four antimicrobials showed that apart from the commonly mutated genes described above, there were no other genes mutated in both datasets [3]. Hence, we must conclude that most mutations that occur during the acquisition of antibiotic resistance were random events that may or may not contribute to the development of resistance. Selection and co-selection afterwards during continued exposure to a specific antimicrobial determine the final list of mutations.

### Functional distribution of mutations

The distribution over functional categories of genes that mutated as a result of exposure to antimicrobials was similar in the various types of cells exposed. This distribution can be related to the mechanisms of action of the particular antibiotic. Both in the dataset on mutations after resistance was built up against two antibiotics [3] and in the present dataset, a specific antibiotic correlates with specific functions. Mutations accompanying resistance to amoxicillin occur in genes coding for proteins involved in cellular processing and signaling and metabolism. This corresponds to the disruption of cell wall construction by beta-lactam antibiotics [55]. Enrofloxacin correlates primarily to genes concerning information storage and processing, due to the inhibition of DNA replication [56], and also to metabolism. Kanamycin mutations are mainly in the area of metabolism, while tetracycline mutations are about evenly divided over cellular processing and signaling and information storage and processing, which also can be understood in the framework of the inhibition of protein synthesis by tetracyclines [57, 58]. The distribution in *L. lactis* was similar to that in *E. coli*.

Prolonged exposure to the beta-lactam antibiotic amoxicillin resulted in the amplification of a chromosomal DNA fragment centered around the *ampC* gene [7]. The multiplication of a 2.5 kb fragment was observed in the aerobically grown MG1655 strain. The amplified segments were larger in the anaerobically grown MG1655 strain (53 kb) and in the mutant Δ*oxyR* both under aerobic (59 kb) and anaerobic (5.5 kb) conditions. This indicates that the same process was taking place, but not in exactly the same manner. The fragment containing the *ampC* gene resembled known plasmid-bound beta-lactam resistance genes and could be transferred to a susceptible *E. coli* strain, that became resistant after this transfer [59]. Plasmids carrying beta-lactam resistance genes are frequently detected in clinical or environmental microorganisms [60]. These observations suggest that the *de novo* development of resistance may play a bigger role in spreading of antimicrobial resistance than previously presumed. A similar duplication of part of the chromosome was also observed as a result of tetracycline exposure. In this case, the copy number was only 2-fold at most and it is doubtful that it played a major role in the acquisition of resistance, also because gene amplification is not known as a tetracycline resistance mechanism.

## Conclusion

The effects of ROS and reactive metabolic byproducts on development of antimicrobial resistance in response to exposure to non-lethal concentrations of antibiotics can be understood by the principle of hormesis. High levels of radical-based stress are lethal, while low levels can increase the rate of acquisition of resistance and thus for the survival of the cell. The role of oxygen in *de novo* acquisition of antibiotic resistance turned out to be indirect as a necessary condition for the generation of ROS. Under anaerobic conditions, the reactive metabolic byproducts seem to function in a similar manner as ROS under aerobic conditions. Still, these stresses caused by reactive compounds remain at a low level when antibiotic concentrations are non-lethal, and the relatively low levels of stress under these conditions accelerate resistance acquisition. By definition, at lethal levels the cells die and no resistance can develop.

## Materials and methods

### Bacterial strains, media and growth conditions

The antibiotic-sensitive wildtype strains *E. coli* MG1655 and *L. lactis* MG1363 were used throughout the study. The *oxyR* gene knockout mutant strain JW3933-3 was obtained from the Keio collection [61], and the kanamycin-resistant cassette was removed using the pCP20 plasmid by FLP Recombination. *E. coli* strains were grown in LB medium or a phosphate-buffered (100 mM NaH_2_PO_4_) defined minimal medium containing 55 mM glucose [62]. *L. lactis* was grown in 10% Lactose M17 broth medium. *E. coli* was grown at 37 °C, *L. lactis* was grown at 30 °C, and both were shaken at 200 rpm. Anaerobic culture DURAN® bottles with a butyl rubber stopper and an open topped screw cap, were filled with medium, inoculated with a syringe. Resazurin was used as oxygen indicator, and these tubes were autoclaved separately. *L. lactis* under aerobic respiration conditions was grown with further addition of heme (Sigma) to a final concentration of 2 µg/mL. Amoxicillin, enrofloxacin, kanamycin, tetracycline, moxifloxacin, and chloramphenicol stock solutions (10 mg/mL) were filter sterilized through a 0.2 µM filter and stored at 4 °C. Fresh antibiotic solutions were made every 3 days.

### Evolution experiments

In order to induce resistance to each antibiotic and test the susceptibility of the strains, the MICs measurement was performed by serial dilution followed by a determination of the initial antibiotic concentrations (Tables 2 and 3).

**Table 2:** Initial MICs of *E. coli* strains

*E. coli* Strains	MG1655	MG1655	Δ*oxyR* Mutant
Medium	LB	Minimal medium	Minimal medium
Amoxicillin	8/16	4	4
Enrofloxacin	4	0.5	1
Kanamycin	64	16	16/32
Tetracycline	8	2	2/4

**Table 3:** Initial MICs of *L. lactis* strains

*L. lactis* Strains	MG1363	MG1363
Medium	LM17	Heme + LM17
Amoxicillin	0.25/0.5	0.25
Moxifloxacin	4	2
Chloramphenicol	2/4	2
Tetracycline	0.5/1	0.5

Evolution experiments inducing resistance were performed as described previously [63]. Briefly, individual colonies of each wild-type strain were isolated and cultured overnight under the specified conditions. Subsequently, fresh culture tubes or anaerobic bottles were inoculated with bacterial volume calculated to result in an initial OD_600_ of 0.1. Antibiotics were added at one-quarter of the MICs. Antibiotic-free groups were cultured under the exact same conditions as control. Following overnight incubation, if the antibiotic-treated group’s OD_600_ exceeded 75% of the control’s OD_600_, a portion of the antibiotic-treated culture was transferred to two new culture systems. These were supplemented with antibiotics at both twice the initial concentration and the original concentration. After another overnight culture, if the higher antibiotic concentration group’s OD_600_ surpassed 75% of the lower concentration group’s OD_600_, the former’s bacterial solution was selected; otherwise, the latter’s solution was chosen. The antibiotic concentration doubling continued until further doubling wasn’t possible and bacterial growth stabilized. Each strain’s evolution experiment was independently performed at least twice. In all experiments, cultures without antibiotic exposure were used as controls. MICs was detected three times a week to monitor resistance.

MICs was measured in 96-well plates in a spectrophotometer plate reader (Thermo Fisher Scientific) at 37^o^C. Each well contains 150 µL final volumes with the OD_600_ of 0.05 bacteria, antibiotics concentrations ranged from 0.25 to 2048 with steps of a factor of two. After overnight culture, the lowest concentration that yielded a final OD_600_ < 0.2 was considered the MICs.

### ROS measurement

To determine the formation of ROS, overnight cultured *E. coli* and *L. lactis* strains made resistant to a specific antibiotic were diluted to OD_600_ of 0.2 and exposed to the highest concentration of these antibiotics that still allowed growth. After administration of the antibiotic, cells were cultured for 3 h at 37 °C (*E. coli*) or 30 °C (*L. lactis*) shaking at 200 rpm. Cell cultures were incubated with 5 µL 10 mM H_2_DCFDA (Sigma) fluorescent dye dissolved in DMSO. Culturing tubes were covered with aluminum foil to prevent exposure to light and incubated for 45 min at the same temperature. After incubation, 1 mL of culture was spun down at 6000 rpm for 5 min, the cell pellet was dissolved in medium. 1.3 µL cell suspension was loaded on a microscope slide glass with 2% agarose mixed with medium. Images were acquired on a Nikon Eclipse Ti microscope with NIS-elements AR software. Fluorescent signal was detected at excitation/emission wavelength of 488/510 nm and was shown in green. Images were processed using Fiji/ImageJ software.

### Whole genome sequencing

The genomic DNA was isolated from the final stable resistant strains by the DNeasy blood and tissue kit (Qiagen). Genomic DNA libraries were generated using the NEBNext Ultra II FS DNA Library Prep kit for Illumina (New England BioLabs) in combination with NEBNext multiplex oligos for Illumina (96 Unique Dual Index Primer Pairs; New England BioLabs) according to the manufacturer’s instructions. Briefly, 500 ng genomic DNA was used as input with a fragmentation time of 5 min, aiming at an insert size distribution of 275–475 bp by following the corresponding size selection option provided in the protocol. The resulting size distribution of the libraries with indexed adapters was assessed using a 2200 TapeStation System with Agilent D1000 ScreenTapes (Agilent Technologies). The libraries were quantified on a QuantStudio 3 Real-Time PCR System (Thermo Fisher Scientific) using the NEBNext Library Quant Kit for Illumina (New England BioLabs) according to the instructions of the manufacturer. The libraries were clustered and sequenced (2 × 150 bp) on a NextSeq 550 Sequencing System (Illumina) using a NextSeq 500/550 Mid Output v2.5 kit (300 cycles) (Illumina). After sequencing, FastQC and MultiQC were used to evaluate the quality of raw reads. BBmerge was used to discover the adapter sequences, which were then imported to Cutadapt to be removed. In order to remove low-quality bases, Trimmomatic was utilized. The removal of optical duplicates was achieved via Clumpify. After mapping the reads to the reference by Bowtie2, GATK was used for marking PCR duplicates. The variant calling was done by Freebayes and used Snpeff to do the variant annotation.

Subsequently, genomic variants were identified by comparing the sequenced genome with the reference genome (NC000913) using the Variant Call Format (vcf) file, analyzed through the Integrative Genomics Viewer (IGV). Furthermore, specific mutated genes were identified by comparing antibiotic-resistant and antibiotic-free strains, excluding common variations. Copy number analysis was performed using cn.MOPS detects larger genomic alterations that result in an abnormal number of copies of one or more genes. Finally, mutated genes were functionally annotated using the Cluster of Orthologous Genes (COG) database and compared across treatment groups.

### Statistical analysis

Statistical analysis was done by using IBM SPASS statistical software. The statistical significance analysis of ROS measurements was investigated using a one-way ANOVA *p < 0.05, **p < 0.001, ***p < 0.0001, Means ± SD. Other experiments not applicable.

## Data Availability

The binary alignment/map (bam) files of the sequenced strains have been deposited in the NCBI database and can accessed at BioProject PRJNA954686 and PRJNA954732.

## Abbreviations

ROS: Reactive oxygen species
RMB: Reactive metabolic byproducts
ETC: Electron transport chain
COG: Clusters of orthologous groups
